# Phytochemical Study of* Tanacetum Sonbolii *Aerial Parts and the Antiprotozoal Activity of its Components

**DOI:** 10.22037/ijpr.2020.1100951

**Published:** 2020

**Authors:** Sahar Mofidi Tabatabaei, Mahdi Moridi Farimani, Samad Nejad-Ebrahimi, Peyman Salehi

**Affiliations:** *Department of Phytochemistry, Medicinal Plants and Drugs Research Institute, Shahid Beheshti University, G. C., Evin, Tehran, Iran.*

**Keywords:** Tanacetum sonbolii, Asteraceae, Flavonoids, Coumarin, Antiprotozoal activity

## Abstract

The genus *Tanacetum* includes some popular endemic species of the flora of Iran, with important medicinal properties. In a project, directed at structurally interesting bioactive metabolites from Iranian endemic species, we studied *Tanacetum sonbolii *Mozaff*.* Eight compounds comprising six phenolic and two terpenoidal compounds were isolated from the ethyl acetate extract of the aerial parts of the plant by normal and reverse phase chromatography*.* Their structures were established mainly by 1D and 2D NMR spectroscopic techniques, including ^1^H-^1^H COSY, HSQC and HMBC methods and confirmed by comparing their NMR data with those reported in the literature. The compounds namely: 2,4-dihydroxy-6-methoxyacetophenone (**1**), apigenin (**2**), 5-desmethylsinensetin (**3**), 5-desmethylnobiletin (**4**), 8-methoxycirsilineol (**5**), scopoletin (**6**), ursolic acid (**7**), and *β*-sitosterol (**8**). *In-vitro* antiprotozoal activity of compounds **1**, **3,** and **5 **were evaluated against *Trypanosoma brucei rhodesiense*, *Trypanosoma cruzi*, *Leishmania donovani* and *Plasmodium falciparum* parasites and also toxicity against rat myoblast (L6) cells. Compound **5** showed promising activity against *T. b. rhodesiense*.

## Introduction

*Tanacetum* is a remarkable genus of the Asteraceae family, comprises about 14 sections and 200 species, native to Europe, Mediterranean region, Western Asia and North America (1). *Tanacetum* species have been used for centuries as valuable herbal remedy in traditional medicine because of their cytotoxic, antimicrobial, antiviral and anti-inflammatory activities (2, 3). *Tanacetum parenthium* (feverfew) which is listed in European Pharmacopeia as a traditional remedy has been used for the treatment of migraine (4, 5). It has been reported that sesquiterpene lactones are the major classes of the secondary metabolites from the genus *Tanacetum*. Also, it has been reported that the flavonoids, coumarins, and triterpenoids are the main classes of compounds in this genus (6–8). In the flora of Iran, the genus *Tanacetum* consists of 36 annual and perennial species, 16 of which are endemic (9)M.V.Agab. & Wagenitz (Asteraceae, Anthemideae. 

*Tanacetum sonbolii* Mozaff. is an endemic species of West Azerbaijan province of Iran. A literature survey showed that there has been no phytochemical study on *T. sonbolii*, apart from an analysis of the essential oil (10). Antioxidant activity, protective effect against hydrogen peroxide-induced oxidative stress in K562 cell line, and effect on PTZ-induced seizures in male mice have been reported for various extracts prepared from different parts of the plant (10–12). As a part of our ongoing research on discovering new and potentially bioactive secondary metabolites from Iranian species (13–15), we investigated the phytochemical compostion of ethyl acetate extract from the aerial parts of *T. sonbolii*. Here we report the isolation and structure elucidation of eight compounds by applying 1D and 2D NMR spectroscopy. Compounds **1**, **3, **and** 5** were tested against *Trypanosoma brucei rhodesiense*, *Trypanosoma cruzi*, *Leish-mania donovani* and *Plasmodium falciparum* parasites, and showed good activities against *T. b. rhodesiense.*

## Experimental

*General experimental procedures*

NMR spectra were mesured at 18 °C on a Bruker Avance III 500 MHz spectrometer, 500.13 MHz for ^1^H, and 125.77 MHz for ^13^C. A 1-mm TXI-microprobe with z-gradient was used for ^1^H-detected experiments. ^13^C NMR spectra were recorded with a 5 mm BBO probe head with z-gradient. The spectra were analyzed using Bruker TopSpin 3.1 software. Deuterated solvents for NMR (100% D) were purchased from Armar Chemicals. HPLC separations were performed on a Knauer HPLC system consisting of a mixing pump 1000 with degasser module, PDA detector, and an autosampler. Knauer Eurospher П 100-5 RP C18 column (5 μm, 4.6 × 250 mm) and SunFire Prep C18 ODB (5 μm, 19 × 50 mm i.d.) columns were used for reversed-phase analytical and semipreparative separations, respectively. The solvents used for extraction and column chromatography were of technical grade and were distilled before use (Emertat, Iran). The solvents used for HPLC were of HPLC grade (Merck, Germany). Silica gel (70–230 mesh) was used for column chromatography, and pre-coated silica gel F254 (20 × 20 cm) plates (Merck, Darmstadt, Germany) for TLC. Anisaldehye (Merck, Germany) was used for visualization of the TLC plates.

*Plant material*

Aerial parts of *Tanacetum sonbolii* were collected from Takab mountains, West-Azerbaijan province, Iran, in June 2015 and identified by Dr. Ali Sonboli. A voucher specimen (MPH-2556) has been deposited in the Herbarium of the Medicinal Plants and Drugs Research Institute of Shahid Beheshti University, Tehran, Iran.

*Extraction and isolation*

The air-dried aerial parts of *T. sonbolii* (1.6 kg) were crushed and extracted with ethyl acetate (5 × 7 L) by maceration at room temperature. The extract was concentrated in vacuum, to afford 60 g of a dark gummy residue. The residue was separated on a silica gel column (70–230 mesh, 6.0 × 120 cm, 850 g) with a gradient of *n*-hexane–EtOAc (100:0 → 0:100) as eluent, followed by increasing concentration of MeOH (up to 100%) in EtOAc. On the basis of TLC analysis (detection at 254 nm, and after spraying anisaldehyde-sulfuric acid reagent), fractions with similar compositions were pooled to yield 21 combined fractions. 

Fraction 2 [eluted with *n*-hexane–EtOAc (80:20)] (1.1 g ) was separated on a silica gel column (70–230 mesh, 2.5 × 45 cm, 150 g) eluted with CH_2_Cl_2_/Me_2_CO (90:10). Fractions of 50 mL were collected and pooled together on the basis of TLC patterns to give seven subfractions (F2_1_-F2_7_). Precipitates of subfraction F2_4_ were recrystallized from Me_2_CO to afford compound **1** (7 mg). From fraction 3 [eluted with *n*-hexane/EtOAc (75:25)] (0.12 g) crude crystals were obtained, which were recrystallized from Me_2_CO to afford compound **8** (12 mg). Fraction 6 [eluted with *n*- hexane/EtOAc (70:30)] (0.15 g) was separated on a silica gel column (70–230 mesh, 2.0 × 64 cm, 110 g) eluted with CH_2_Cl_2_/Me_2_CO (80:20). Fractions of 50 mL were collected, and combined to five subfractions (F6_1_–F6_5_) on the basis of TLC patterns. Recrystallization of subfraction F6_3_ afforded compound **7 **(20 mg). Fraction 13 [eluted with *n*-hexane–EtOAc (60:40)] (0.09 g) was separated by preparative HPLC (MeCN/ H_2_O, 50:50, v/v) to yield **2** (0.8 mg), **3** (0.8mg) and **4** (0.4mg). Fraction 15 [eluted with *n*-hexane–EtOAc (40:60)] (0.10 g) was separated by preparative HPLC (MeCN/ H_2_O, 40:60, v/v) to yield compounds **5** (3 mg) and **6** (2 mg).

*In-vitro Biological Testing*

The *in-vitro* activities against the protozoan parasites *Trypanosoma brucei rhodesiense *(STIB900) bloodstream forms, *Trypanosoma cruzi *(Tulahuen C4 LacZ) intracellular amastigotes, *Leishmania donovani* (MHOM-ET-67/L82) axenically grown amastigotes, and *Plasmodium falciparum *(NF54) erythrocytic stage and also the cytotoxicity against L6 cells were tested according to the literature procedures (16, 17).

*Statistical analysis*

All measurements were expressed as the mean ± standard deviations (SD) in triplicate manner. Excel 2010 was employed for analyzing data. The IC_50_ values were calculated by linear regression from the sigmoidal dose inhibition curves using SoftmaxPro software. The selectivity index was calculated as IC_50_ for L-6 cells/IC_50_ for parasites.

## Results and Discussion

**Figure 1:**
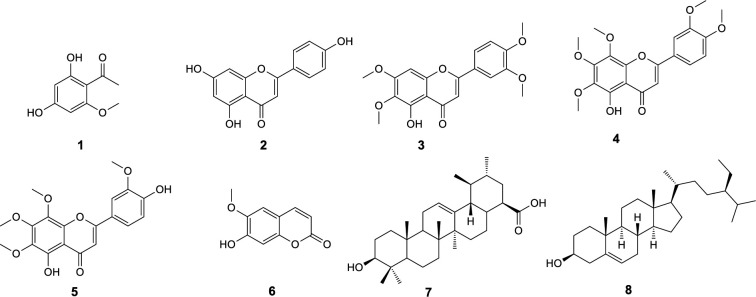
The structures of compounds **1-8**

Purification processes on ethyl acetate extract obtained from aerial parts of *T. sonbolii* via chromatography on normal and reverse phase silica gel columns as well as recrystallization led to isolation and identification of eight known compounds. Structure elucidation was accomplished by 1D and 2D NMR spectra (COSY, HMQC-DEPT, and HMBC) and confirmed by comparing their NMR data with those reported in the literature. The compounds including six phenolics comprise four flavone type, apigenin (**2**) (18), 5-desmethylsinensetin (**3**) (19), 5-demethylnobiletin (**4**) (20), 8-methoxycirsilineol (**5**) (20), a cumarin, scopoletin (**6**) (21), and a phenylethanone, 2,4-dihydroxy-6-methoxyacetophenone (**1**) (22). Moreover, a triterpenoid, ursolic acid (**7**) (23), and an steroidal compound, *β*-sitosterol (**8**) (24) were also isolated (Figure 1). Compounds **1 **(25), **2 **(26), **3 **(27), **4 **(28), **6 **(29)**, **and **8** (30) have been previously isolated from other *Tanacetum* species; however the compounds **5 **and** 7** are reported here from this genus for the first time.

*In-vitro* antiprotozoal activity of compounds **1**,** 3, **and** 5** were studied against *T. b. rhodesiense*, *T. cruzi*, *L. donovani,* and *P. falciparum*. The compounds showed good inhibition against *T. b. rhodesiense*. Compound **1 **inhibited the growth of the parasite equal to 95.2% at the concentration of 10 µg/mL, while Melarsoprol as the reference compound showed 100% inhibition. Also, compound **3 **and** 5 **showed considerable inhibition equal to 99.6% and 96.8% at 10 µg/mL concentration, respectively. Accordingly, these compounds were considered for further study to evaluate the IC_50_ values against *T. b. rhodesiense* and cytotoxicity against L6 cells. The results are presented in Table 1; Compound **5** exhibited the most inhibitory activity against *T. b. rhodesiense* with IC_50_ value of 6.1 μM and selectivity index (SI) equal to 5.4.

In the past few years the class of phenolics specially flavonoids and cumarins have attracted attention of scientists as good antiprotozoal agents, particularly against *T. brucei rhodesiense*, the causative agent of the East African form of Human Africa Trypanosomiasis (HAT) (31–35). Flavonoid structures similar to those we reported here such as apigenin (36)”ISSN” : “15206025”, “PMID” : “25768915”, “abstract” : “Leishmaniasis is an important neglected disease caused by protozoa of the genus Leishmania that affects more than 12 million people worldwide. Leishmaniasis treatment requires the administration of toxic and poorly tolerated drugs, and parasite resistance greatly reduces the efficacy of conventional medications. Apigenin (1, sinensetin (35), and cirsilineol (37) and also the flavonoids with more than two methoxy groups have shown considerable activities against several protozoan parasites (33, 38, 32, 39). In a previous study, 5-desmethylsinensetin (compound **3**) showed IC_50_ values of 0.2 mg/mL against *T. cruzi epimastigotes*, 78.7 mg/mL against *T. cruzi amastigotes,* and 37.0 mg/mL against *L. braziliensis promastigotesso* (35).

*Spectral data of the isolated compounds*

*2, 4-dihydroxy- 6-methoxyacetophenone (* ***1*** *)*

Colourless crystals; ^1^H NMR (DMSO, 500.13 MHz): *δ* 2.50 (3H, s, CH_3_), 3.82 (3H, s, OMe), 5.87 (1H, brd s, H-3), 5.97 (1H, brd s, H-5); ^13^C NMR (DMSO, 125 MHz): *δ* 33.0 (CH_3_), 56.2 (OMe), 91.7 (C-5), 96.0 (C-3), 105.0 (C-1), 163.8 (C-6), 165.6 (C-4), 166.8 (C-2), 202.6 (CO).

*Apigenin (* ***2*** *)*

yellow powder;^ 1^H NMR (500.13 MHz, CDCl_3_), *δ* (ppm): 6.16 (1H, s, H-6), 6.45 (1H, s, H-8), 6.68 (1H, s, H-3) , 6.93 (2H, d, *J*=8.6 Hz, H-3’, 5’), 7.88 (2H, d, *J*=8.6 Hz, H-2’, 6’), 12.96 (1H, s, OH). for ^13^C NMR (125.77 MHz, CDCl_3_), *δ* (ppm): 93.9 (C-8), 98.8 (C-6), 102.7 (C-3), 104.6 (C-10), 115.9 (C-3’, 5’),120.6 (C-1’), 128.1 (C-2’), 129.4(C-6’), 156.5 (C-5), 161.4 (C-9), 162.0 (C-4’), 163.2(C-7), 165.3(C-2), 181.9 (C-4).

*5-desmethylsinensetin (* ***3*** *)*

Yellow powder;^ 1^H NMR (500.13 MHz, CDCl_3_), *δ* (ppm): 3.88, 3.89, 3.90, 3.91(3H, s, OMe 6,7, 3’, 4’), 6.54 (1H, s, H-3), 6.58 (1H, s, H-8), 6.97 (1H, d, *J* = 8.5 Hz, H-5′), 7.33 (1H, d, *J* = 2.5 Hz, H-2′), 7.52 (1H, dd, *J* = 8.5, 2.5 Hz, H-6′), 12.38 (1H, s, OH). for ^13^C NMR (125.77 MHz, CDCl_3_), *δ* (ppm): 55.8 (OMe 7), 56.1 (OMe 3’, 4’), 60.8 (OMe 6), 90.8 (C-8), 104.5 (C-3), 106.4 (C-10), 109.1 (C-2′),111.5 (C-5′), 120.3 (C-6′), 124.5 (C-1’), 132.9 (C-6), 149.6 (C-3′), 152.6 (C-4′), 153.3 (C-5), 153.5 (C-9), 159 (C-7), 163.6 (C-2), 182.0 (C-4).

*5-demethylnobiletin* *(****4****)*

Yellow powder;^ 1^H NMR (500.13 MHz, CDCl_3_), *δ* (ppm): 3.80, 3.82, 3.84, 3.86 (3H, s, OMe 6, 7, 8, 3’, 4’), 6.48 (1H, s, H-3), 6.90 (1H, d, *J*=1.5 Hz, H-2’) , 7.30 (1H, d, *J*=6.8 Hz, H-5’), 7.59 (1H, dd, *J*=1.5 Hz, 6.8 Hz, H-6’), 12.50 (1H, s, OH). for ^13^C NMR (125.77 MHz, CDCl_3_), *δ* (ppm): 56.6 (OMe 3’), 57.1 (OMe 4’ ), 60.1 (OMe 8), 61.2 (OMe 6), 61.4 (OMe 7), 104.5 (C-3), 107.4 (C-10), 111.7 (C-2’), 109.5 (C-5’), 121.2 (C-6’), 123.6 (C-1’), 132.5 (C-8), 136.8 (C-6), 146.1 (C-9), 150.9 (C-4’), 149.5 (C-5), 149.5 (C-3’), 152.4 (C-7), 163.6 (C-2), 182.1 (C-4).

*8-methoxycirsilineol (* ***5*** *)*

Yellow crystals; ^1^H NMR (500.13 MHz, CDCl_3_), *δ* (ppm): 3.80 (3H, s, OMe 7), 3.82 (3H, s, OMe 8), 3.84 (3H, s, OMe 3’), 3.86 (3H, s, OMe 6), 6.40 (1H, s, H-3), 6.85 (1H, d, *J *= 1.7 Hz, H-2’) , 7.58 (1H, d, *J *= 6.5 Hz, H-5’), 7.59 (1H, dd, *J *= 1.7 Hz, 6.5 Hz, H-6’), 12.54 (1H, s, OH). for ^13^C NMR (125.77 MHz, CDCl_3_), *δ* (ppm): 56.6 (OMe 3’), 57.1 (OMe 7 ), 60.1 (OMe 6), 61.2 (OMe 8), 104.5 (C-3), 105.4 (C-10), 112.0 (C-2’), 117.1 (C-5’), 121.2 (C-6’), 123.6 (C-1’), 132.5 (C-8), 136.8 (C-6), 145.2 (C-9), 146.9 (C-4’), 149.5 (C-5), 149.2 (C-3’), 152.4 (C-7), 163.6 (C-2), 182.1 (C-4).

*scopoletin (****6****)*

Yellow crystals; ^1^H NMR (500.13 MHz, CDCl_3_), *δ* (ppm): 3.88 (3H, s, OMe), 6.18 (1H, d, *J*= 9.2 Hz, H-3), 6.77 (1H, s, H-5), 6.84 (1H, s, H-8), 7.50 (1H, d, *J *= 9.2 Hz, H-4) for ^13^C NMR (125.77 MHz, CDCl_3_), *δ* (ppm): 56.1 (OMe), 102.7 (C-8), 110.2 (C-5), 111.3 (C-10), 113.4 (C-3), 143.5 (C-4), 144.0 (C-7), 145.9 (C-6), 150.0 (C-9), 161.9 (C-2).

Ursolic acid *(****7****)*

White powder; ^1^H NMR (500.13 MHz, CDCl_3_), *δ* (ppm): 0.68 (3H, s, Me-23), 0.75 (3H, s, Me-26), 0.81 (3H, d, *J* = 6.25 Hz, Me-29), 0.87 (3H, s, Me-25), 0.89 (3H, s, Me-24), 0.92 (3H, d, *J* = 6.5 Hz, Me-30), 1.04 (3H, s, Me-27), 2.10 (1H, d, *J* = 11.25 Hz, H-18),3.00 (1H, m, H-3), 4.31 (1H, brs, D_2_O exchangeable, OH), 5.13 (1H, m, H-12). for ^13^C NMR (125.77 MHz, CDCl_3_), *δ* (ppm): 16.2 (C-24), 16.7 (C-25), 17.8 (C-26), 17.9 (C-29), 18.9 (C-6), 21.9 (C-30), 23.7 (C-11), 24.1 (C-27), 24.7 (C-16), 27.8 (C-2), 28.4 (C-15), 29.1 (C-23), 31.1 (C-21), 33.6 (C-7), 37.2 (C-22), 37.4 (C-10), 39.2 (C-4), 39.2(C-1), 39.3 (C-20), 39.4 (C-19), 40.0 (C-8), 42.5 (C-14), 47.7 (C-17), 47.9 (C-9 ), 53.2 (C-18), 55.6 (C-5), 77.7 (C-3), 125.4 (C-12), 139.0 (C-13), 179.1 (C-29).

*β*-sitosterol *(****8****)*

Colorless crystals; ^1^H NMR (500.13 MHz, CDCl_3_), *δ* (ppm): 0.68 (3H, s, H-18), 0.81 (3H, brd s, H-26), 0.82 (3H, brd s, H-27), 0.84 (3H, brd s, H-24b), 0.92 (3H, d, *J* = 6.7 Hz, H-21), 1.01 (3H, s, H-19), 3.45 (1H, m, H-3), 5.37 (1H, m, H-6). for ^13^C NMR (125.77 MHz, CDCl_3_), *δ* (ppm): 12.0 (C-18), 12.2 (C-29), 18.8 (C-21), 19.0 ( C-26), 19.3 (C-27), 19.8 (C-19), 21.1 (C-11), 23.2 (C-28), 25.9 (C-15), 26.3 (C-16), 26.4 (C-23), 29.2 (C-25), 30.4 (C-2), 31.8 (C-7), 32.0 (C-8), 33.9 (C-22), 36.1 (C-10), 36.2 (C-20), 37.4 (C-1), 39.8 (C-12), 41.8 (C-4), 42.7 (C-13),45.9 (C-24), 50.8 (C-9), 56.2 (C-17), 56.5 (C-14), 71.6 (C-3), 121.8 (C-6), 140.8 (C-5).

**Table 1:** *In-vitro* activity of compounds **1**, **3** and **5** against *T. b. rhodesiense* (STIB 900) and cytotoxicity on L6 Cells [IC_50_ (μM)].

Compound	*T. b. rhodesiense* ^a^	L6 cells
**1**	>200	**-**
**3**	14.3 ± 0.1 (5.2)^b^	74.1 ± 0.1
**5**	6.1 ± 0.1 (5.4)^b^	32.6 ± 0.2
**Melarsoprol**	0.003 ± 0.01	**-**
**Podophyllotoxin**	-	0.005 ± 0.01

^a^ Average of three independent assays. ^b^ Selectivity index (SI): IC_50_ in L6 cells divided by IC_50_ in the title parasite strain.

## Conclusion

In the present study, eight known compounds were isolated from the aerial parts of *Tanacetum sonbolii*. Their structures were elucidated by means of extensive 1D, 2D NMR spectroscopy. Also the *in-vitro* antiprotozoal activity of the compounds was studied. Compounds **1**, **3,** and **5** were the active constituents for growth inhibition of *T. brucei rhodesiense *at 10 µg/mL concentration and the most active compound was compound **5** with IC_50_ value of 6.1 μM and selectivity index of 5.4.

## References

[1] Bhat G, Masood A, Ganai BA, Hamza B, Ganie S, Shafi T, Idris A, Shawl A, Tantry A (2016). Gracilone, a new sesquiterpene lactone from Tanacetum gracile (Tansies). Nat. Prod. Res..

[2] Gören N, Arda N, Çaliskan Z (2002). Chemical characterization and biological activities of the genus Tanacetum (Compositae). Stud. Nat. Prod. Chem..

[3] Long C, Sauleau P, David B, Lavaud C, Cassabois V, Ausseil F, Massiot G (2003). Bioactive flavonoids of Tanacetum parthenium revisited. Phytochemistry.

[4] Wichtl M (2004). Herbal Drugs and Phytopharmaceuticals a Handbook of Practiceon a Scientific Basis.

[5] European Pharmacopoeia (2008). Tanaceti parthenii herba 01/2008:1516.European Directorate for the Quality of Medicines and Health Care (EDQM. Council of Europe Strasbourg.

[6] Cambie RC, Lal AR, Ahmad F (1990). Sesquiterpenes from Heritiera ornithocephala. Phytochemistry.

[7] González AG, Barrera JB, Méndez JT, Sanchez ML, Eiroa Martínez JL (1992). Sesquiterpene lactones and other constituents of Tanacetum species. Phytochemistry.

[8] Marzouk MM, Mohamed TA, Elkhateeb A, El-toumy SA, Hegazy MEF (2016). Phenolics from Tanacetum sinaicum (Fresen) Delile ex bremer & Humphries (Asteraceae). Biochem. Syst. Ecol..

[9] Kazemi M, Sonboli A, Zare Maivan H, Kazempour Osaloo S (2014). A taxonomic reassessment of the Tanacetum aureum (Asteraceae, Anthemideae) species group: Insights from morphological and molecular data. Turk. J. Botany.

[10] Firozy M, Talebpour Z, Sonboli A (2012). Essential oil composition and antioxidant activities of the various extracts of Tanacetum sonbolii Mozaff (Asteraceae) from Iran. Nat. Prod. Res..

[11] Esmaeili MA, Sonboli A, Ayyari Noushabadi M (2010). Antioxidant and protective properties of six Tanacetum species against hydrogen peroxide-induced oxidative stress in K562 cell line: A comparative study. Food Chem..

[12] Mohammad M, Zadeh, Naderi F, Azhdari H, Zarmehri, Sonboli A, Soufiabadi M, Mohammad zadeh M (2012). The Effect of Tanacetum sonbolii Hydroalcholic Extract on PTZ-Induced Seizures in Male Mice. J. Med. Plants.

[13] Mofidi Tabatabaei S, Salehi P, Moridi Farimani M, Neuburger M, De Mieri M, Hamburger M, Nejad-ebrahimi S (2017). A nor-diterpene from Salvia sahendica leaves. Nat. Prod. Res..

[14] Moridi Farimani M, Miran M (2014). Labdane diterpenoids from Salvia reuterana. Phytochemistry.

[15] Farimani MM, Mazarei Z (2014). Fitoterapia Sesterterpenoids and other constituents from Salvia lachnocalyx Hedge. Fitoterapia.

[16] Orhan I, Şener B, Kaiser M, Brun R, Tasdemir D (2010). Inhibitory activity of marine sponge-derived natural products against parasitic protozoa. Mar. Drugs.

[17] Schmidt T, Nour A, Khalid S, Kaiser M, Brun R (2009). Quantitative Structure ‒ Antiprotozoal Activity Relationships of Sesquiterpene Lactones. Molecules.

[18] Faizi S, Siddiqi H, Naz A, Bano S, Lubna (2010). Specific deuteration in patuletin and related flavonoids via keto - Enol tautomerism: Solvent- and temperature-dependent 1H-NMR studies. Helv. Chim. Acta.

[19] Awad BM, Habib ES, Ibrahim AK, Wanas AS, Radwan MM, Helal MA, Ahmed S (2017). Cytotoxic activity evaluation and molecular docking study of phenolic derivatives from Achillea fragrantissima (Forssk) growing in Egypt. Med. Chem. Res..

[20] Hamdan D, El-Readi MZ, Tahrani A, Herrmann F, Kaufmann D, Farrag N, El-Shazly A, Wink M (2011). Chemical composition and biological activity of Citrus jambhiri Lush. Food Chem..

[21] Wu Y Bin, Zheng CJ, Qin LP, Sun LN, Han T, Jiao L, Zhang Q, Wu J (2009). Antiosteoporotic activity of anthraquinones from Morinda officinalis on osteoblasts and osteoclasts. Molecules.

[22] Dagne E, Steglich W (1984). Knipholone: a unique anthraquinone derivative from Kniphofia foliosa. Phytochemistry.

[23] Wu L, Wang G, Shen T, Qiang Q, Xue Q, Chen M, Zhang J, Luo Y, Hong Y, Ling si C, Hu W (2018). Chemical constituents of leaves of Mahonia bealei. Chem. Nat. Comp..

[24] Terra WDS, Vieira IJC, Braz-Filho R, De Freitas WR, Kanashiro MM, Torres MCM (2013). Lepidotrichilins A and B, new protolimonoids with cytotoxic activity from Trichilia lepidota (meliaceae). Molecules.

[25] Gören N, Tahtasakal E (1994). Constituents of Tanacetum densum subsp. Eginense. Phytochemistry.

[26] Williams C, Harborne JB, Geiger H, Robin J, Hoult S (1999). The flavonoids of Tanacetum parthenium and T vulgare and their anti-inflammatory properties. Phytochemistry.

[27] Gören N, Kirmizigül S, Zdero C (1997). A farnesol derivative from Tanacetum aucheranum. Phytochemistry.

[28] Hussain J, Munir M, Hassan Z, Bano N, Arshad S, Ahmad VU (2010). Tanacetamide D: A new ceramide from Tanacetum artemisioides. Helv. Chim. Acta.

[29] Martinez LE, Productos C De, Orglnicos N, Gonzblez A, Laguna L, Islands C (1989). Sesquiterpene lactones from Tanacetum Ferulaceum Antoni. Phytochemistry.

[30] Mahmood U, Kaul VK, Singh B (2002). Sesquiterpene and long chain ester from Tanacetum longifolium. Phytochemistry.

[31] Tasdemir D, Kaiser M, Brun R, Yardley V, Schmidt TJ, Tosun F, Ruedi P (2006). Antitrypanosomal and antileishmanial activities of flavonoids and their analogues: in-vitro, in-vivo, structure-activity relationship, and quantitative structure-activity relationship studies. Antimicrob. Agents Chemother..

[32] Nour AMM, Khalid SA, Kaiser M, Brun R, Abdalla WE, Schmidt TJ (2010). The antiprotozoal activity of methylated flavonoids from Ageratum conyzoides L. J. Ethnopharmacol..

[33] Taleb-Continil SH, Salvador MJ, Balanco JMF, Albuquerque S, De Oliveira DCR (2004). Antiprotozoal Effect of Crude Extracts and Flavonoids Isolated from Chromolaena hirsuta (Asteraceae). Phyther. Res..

[34] Kaur R, Dogra NK (2014). A Review on Traditional Uses , Chemical Constituents and Pharmacology of Ageratum conyzoides L ( Asteraceae ). Int. J. Phaemaceutical Biol. Arch..

[35] Beer MF, Frank FM, Germán Elso O, Ernesto Bivona A, Cerny N, Giberti G, Malchiodi L, Martino VS, Aronso MR, Sulsen VP, Cazorla SI (2016). Trypanocidal and leishmanicidal activities of flavonoids isolated from Stevia satureiifolia var. satureiifolia. Pharm. Biol..

[36] Fonseca-Silva F, Canto-Cavalheiro MM, Menna-Barreto RFS, Almeida-Amaral EE (2015). Effect of Apigenin on Leishmania amazonensis Is Associated with Reactive Oxygen Species Production Followed by Mitochondrial Dysfunction. J. Nat. Prod..

[37] Tasdemir D, Tierney M, Sen R, Bergonzi MC, Demirci B, Bilia AR, Baser K, Brun R, Chatterjee M (2015). Antiprotozoal Effect of Artemisia indica Extracts and Essential Oil. Planta Med..

[38] Nwodo N, Okoye F, Lai D, Debbab A, Kaiser M, Brun R, Proksch P (2015). Evaluation of the in-vitro trypanocidal activity of methylated flavonoid constituents of Vitex simplicifolia leaves. BMC Complement. Altern. Med..

[39] M RME, Mendoza AJ, Arreola GR, Ordaz PC (2010). Flavonoids with antiprotozoal activity. Rev. Mex. Ciencias Farm..

